# 基于串联质量标记的帕金森病血浆及血浆外泌体定量蛋白质组学分析

**DOI:** 10.3724/SP.J.1123.2022.12022

**Published:** 2023-12-08

**Authors:** Yuan ZHAO, Xin LIU, Yidan ZHANG, Jian ZHANG, Xiang LIU, Guofeng YANG

**Affiliations:** 1.河北医科大学第二医院老年病科,河北石家庄050051; 1. Department of Geriatrics, Second Hospital of Hebei Medical University, Shijiazhuang 050051, China; 2.河北医科大学第二医院神经外科,河北石家庄050051; 2. Department of Neurosurgery, Second Hospital of Hebei Medical University, Shijiazhuang 050051, China

**Keywords:** 串联质量标记, 定量蛋白质组学, 帕金森病, 外泌体, 血浆标志物, tandem mass tag (TMT), quantitative proteomics, Parkinson’s disease (PD), exosomes, plasma biomarkers

## Abstract

外泌体是一类可由各种细胞在生理和病理条件下释放的细胞外囊泡,其携带了多种生物活性分子,是疾病标志物的良好载体。目前,帕金森病(Parkinson’s disease, PD)的诊断主要依靠临床表现,缺乏客观的疾病诊断标志物。因此,新型外周血特异性标志物的开发将有助于PD的早期筛查与诊疗。在本研究中,选取PD患者与正常对照人群的血浆及血浆外泌体作为研究对象,采用基于串联质量标记(tandem mass tag, TMT)的液相色谱-串联质谱(LC-MS/MS)技术对其进行定量蛋白质组学分析,在血浆和血浆外泌体样品中分别定量到724和611个蛋白质。采用基因集富集分析(gene set enrichment analysis, GSEA)对定量到的所有蛋白质进行生物学信息分析,以了解蛋白质的基因本体论(gene ontology,GO)、京都基因和基因组百科全书(Kyoto Encyclopedia of Genes and Genomes,KEGG)通路富集情况。根据细胞组分(cellular component, CC)分析,PD和正常对照组血浆样本中的差异表达蛋白质主要定位于细胞核中,血浆外泌体的差异表达蛋白质主要定位于细胞质中。与血浆差异表达蛋白质相关的分子功能(molecular function, MF)主要涉及RNA、DNA结合及补体结合等过程;而血浆外泌体差异表达蛋白质的分子功能主要表现为抗氧化作用、氧化还原酶活性调节作用等。由此可见,血浆外泌体中的差异表达蛋白质富集到的分子功能更具有疾病特异性。基于|log_2_差异倍数(FC)|>0.26和统计学意义(*P*-value, *P*)<0.05,在PD血浆样本中共筛选出11个差异表达蛋白质,其中5个蛋白质表达上调,6个蛋白质表达下调;在血浆外泌体样本中,共筛选出13个差异表达蛋白质,其中6个蛋白质表达上调,7个蛋白质表达下调。本研究通过分析血浆及血浆外泌体样本的蛋白质组学信息来探究PD的发病机制,并通过比较发现,外泌体样本能够获得更多的差异表达蛋白质和生物学信息,为发现新型PD生物标志物和治疗靶点提供了新思路。

## Abstract (en)

The cardinal clinical features of Parkinson’s disease (PD), a common neurodegenerative disease, include the irreversible impairment of movement coordination, such as tremors, gait rigidity, bradykinesia, and hypokinesia. Although various factors are associated with the pathological changes in PD, such as oxidative stress, mitochondrial dysfunction, and neuroinflammation, the availability of treatments to retard PD progression is limited. Therefore, novel biomarkers for PD diagnosis and therapeutic targets are urgently needed. The diagnosis of PD mainly depends on its clinical manifestations and has an error rate of approximately 20%. Studies have shown that *α*-synuclein (*α*-syn) levels are significantly increased in the cerebrospinal fluid of patients with PD; however, the invasive nature of lumbar puncture restricts further studies on its clinical applications. Hence, the development of novel peripheral blood markers would be helpful for the early diagnosis of PD. Exosomes are extracellular vesicles (EVs) released by various cell types under physiological and pathophysiological conditions. Because exosomes carry a variety of bioactive molecules, they play a key role in biological processes such as intercellular communication and the immune response. Central nervous system (CNS)-derived exosomes can be detected in the cerebrospinal and peripheral body fluids of patients with PD, and their contents are altered during the disease process, rendering them an attractive biomarker resource. Therefore, a comprehensive and high-throughput investigation of the plasma and its exosomes may enhance our understanding of PD. In this study, we isolated exosomes from plasma using standard differential centrifugation and performed tandem mass tag (TMT)-labeled quantitative proteomic analysis of plasma and plasma exosome samples from healthy individuals and patients with PD using liquid chromatography-tandem mass spectrometry (LC-MS/MS). A total of 724 proteins were quantified in the plasma samples, and 611 proteins were screened from the exosome samples. Among these 611 proteins, 413 were found in the Exosomal Protein Database (Exocarta). Using |log_2_FC|>0.26 and *P*-value (*P*)<0.05 as the cutoff, five upregulated and six downregulated proteins were identified in the plasma samples of the PD group compared with the healthy group. In the plasma exosome samples, compared with the healthy group, the PD group showed six upregulated and seven downregulated proteins. Gene ontology (GO) and Kyoto Encyclopedia of Genes and Genomes (KEGG) analyses were conducted based on gene set enrichment analysis (GSEA). GO-cellular component (CC) analysis revealed that plasma-enriched proteins were mainly located in the nucleus whereas plasma exosome-enriched proteins were mainly located in the cytoplasm. According to the GO-molecular function (MF) analysis, the MFs of differentially expressed proteins in the plasma were mainly enriched in RNA, DNA binding, and complement binding. By contrast, the molecular functions of differentially expressed proteins derived from plasma exosomes were enriched in antioxidant activity, oxidoreductase activity, and peroxide acceptor activity. We then analyzed the enriched KEGG pathways of differentially expressed proteins derived from the plasma and plasma exosome samples. The enrichment pathways of differentially expressed proteins in the plasma samples included the lysosome pathway, cellular senescence, and protein processing in the endoplasmic reticulum. By contrast, the enrichment pathways of differentially expressed proteins in the plasma exosome samples included chemokine signaling and cytokine receptor interactions. Finally, we assessed the functions of some exosomal proteins in PD to elucidate their potential for PD diagnosis and treatment. Significant differences were observed between the plasma and plasma exosome protein profiles, and the functions of differentially expressed proteins in plasma exosomes were strongly related to the pathology of PD. Our study provides a reference for identifying the potential biomarkers and therapeutic targets of PD.

帕金森病(Parkinson’s disease, PD)是最常见的、能够引起老年人运动障碍的神经系统退行性疾病,其病因尚不明确。随着人口的老龄化,PD在全球出现增长的趋势,随之带来一系列的家庭、医学及社会问题。PD主要病变在黑质致密带、迷走神经背核、蓝斑等部位,其典型病理变化是*α*-突触核蛋白(*α*-syn)的聚集及尾状核、壳核中多巴胺含量的减少^[1]^。当PD患者出现临床症状时,黑质多巴胺能神经元的凋亡比例至少为50%以上,纹状体多巴胺含量减少80%以上^[2]^。由此可见,PD患者具有临床症状与神经损伤的不平行性,神经损伤早于临床症状的出现。目前PD的临床诊断仍以症状诊断为主,多数患者在确诊时已出现较为明显的运动障碍。因此,开发PD的早期诊断标志物对于开展早期治疗、改善病人预后等具有重要意义。

外泌体(exosomes)是一种直径为50~200 nm且具有双层脂质膜结构的细胞外囊泡^[2]^。外泌体携带了大量来源细胞的蛋白质、核苷酸等生物分子,在细胞间通讯和免疫反应等生物过程中发挥着重要作用^[3⇓⇓-6]^。外泌体参与多种神经系统生理过程(包括物质转运、髓鞘形成和受损轴突的再生等),调节各种信号功能,并与神经系统退行性疾病相关^[7⇓-9]^。血浆中存在多种细胞来源外泌体,其中包括神经源性外泌体。研究表明,血浆外泌体携带了能够反映中枢神经系统病变的病理性生物分子^[10]^。目前,越来越多的研究开始关注PD的外泌体生物标志物,例如通过血浆外泌体的微小核糖核酸(miRNA)逆转录定量聚合酶链反应(RT-qPCR)分析发现,与对照组相比,PD组外泌体中的miR331-5p水平明显升高,而miR-505水平则明显降低;且miR331-5p和miR-505具有较好的PD诊断价值及预测能力^[11]^。不仅如此,有研究表明,血浆外泌体的朊病毒蛋白质水平上升与PD患者认知能力下降显著相关(统计学意义(*P*-value, *P*)<0.05)^[12]^。由此可见,外泌体中可能承载了更多具有特异性的生物学信息,是中枢神经系统疾病生物标志物的良好载体。

蛋白质组学技术是研究生物标志物的重要手段^[13]^,血浆和血浆外泌体中含有不同的蛋白质谱,二者均具有作为蛋白质组学分析样本的潜力。蛋白质通常需要与其他蛋白质相互作用才能发挥生物功能,因此蛋白质组学具有整体性。基因组学发现了一些与疾病相关的遗传突变,但多数疾病的发生可能还与后期蛋白质的表达量、修饰及相互作用的改变相关,因此蛋白质组学研究更有助于揭示这些疾病的本质^[14,15]^。已有研究表明,通过蛋白质组学技术鉴定到的血浆差异表达蛋白质可以区分PD患者与健康对照者(healthy control, HC)^[14]^,但关于血浆外泌体的蛋白质组学研究仍较少。因此,本研究通过差速超速离心法分离HC和PD患者的血浆外泌体,使用基于串联质量标记(tandem mass tag, TMT)的液相色谱-串联质谱(LC-MS/MS)技术分别对两组样本的血浆和血浆外泌体进行定量蛋白质组学分析,从整体水平上研究PD的蛋白质变化,探究血浆及血浆外泌体样本在组学分析中的差异,筛选新型PD外周血标志物;之后对定量到的蛋白质进行基因集富集分析(gene set enrichment analysis, GSEA),分析定量蛋白质的富集通路及细胞定位等情况,所获得的生物学信息有助于在蛋白质水平上了解PD的病理过程及分子机制。

## 1 实验部分

### 1.1 仪器、试剂与材料

EASY-nLC 1200液相色谱系统、PepMap RSLC C18色谱柱(25 cm×75 μm, 2 μm)、Orbitrap Fusion Lumos融合荧光闪烁三合一质谱仪、SPD120真空离心蒸发浓缩器(美国Thermo Fisher Scientific公司); optimal-90K超速离心机、70 Ti转子(美国Beckman Coulter公司); Tecnai G2 20双透射电镜(200 kV,美国FEI公司);ZetaView PMX 110纳米颗粒跟踪分析仪(德国Particle Metrix公司)。

10标TMT试剂、Pierce Bicinchoninic Acid蛋白质试剂盒、色谱级乙腈和甲醇、Bradford蛋白质浓度检测试剂盒、Pierce^TM^比色法定量检测试剂盒(美国Thermo Fisher Scientific公司);高选择性Top14高丰度蛋白损耗树脂(Cat No. A36370,美国Sigma-Aldrich公司); 200目碳层铜网(北京中科科仪股份有限公司);色谱级甲酸(德国CNW公司); 0.22 μm孔径过滤器、聚偏氟乙烯膜、化学发光辣根过氧化物酶(HRP)底物(美国Millipore公司); ALG-2相互作用蛋白X (Alix,货号2171S)、钙网蛋白(Calreticulin,货号12238T)一抗均购于美国Cell Signaling公司;脂筏标记蛋白(Flotillin-1,货号610820)购于美国BDBiosciences公司;二抗HRP标记山羊抗兔免疫球蛋白G (货号4050-05,美国Southern Biotech公司)。

### 1.2 血浆样本采集

本研究选取2019年9月至2020年7月就诊于河北医科大学第二医院老年病科的9名PD患者及9名HC为研究对象。PD患者的诊断均符合2016版中国帕金森病诊断和拟诊标准,病程达1年以上;临床排除标准:(1)继发性帕金森综合征患者,(2)帕金森叠加综合征患者,(3)有严重心、肝、肾、胃肠道功能障碍者,(4)提供病史不详细者,(5)近1个月内有感染者,(6)患有免疫系统疾病者,(7)有慢性感染性疾病者,(8)近1个月内使用过消炎药、免疫调节药者,(9)有恶性肿瘤病史者,(10)近期有外伤或头颅手术者。样本采集前,HC和PD患者均签署了书面知情同意书,受试者资料如表1所示。本研究已获河北医科大学第二医院机构伦理委员会批准(批准文号:2022-R036)。

**表1:** 受试者的人口统计学及临床特征

Character	HC (*n*=9)	PD (*n*=9)
Gender (male/female)	5/4	4/5
Age (year)	70±1.91	65±1.56
BMI / (kg/m^2^)	21±0.30	20±0.50
PD duration (Year)	-	6±0.88
UPDRS-Ⅲ score	0	22±1.89
Hoehn and Yahr scale	-	2.86±0.21
Unmedicated (N)	9	1
Levodopa (N)	-	3
DA (N)	-	2
Levodopa+DA (N)	-	2
Levodopa+DA+other	-	1
treatments (N)		

HC: healthy control; PD: Parkinson’s disease; BMI: body mass index; UPDRS-Ⅲ: unified Parkinson’s disease rating scale Ⅲ-motor scores; Hoehn and Yahr scale: a commonly used system for describing how the symptoms of Parkinson’s disease progress; N: number; -: uninvolved; DA: dopamine receptor agonists.

### 1.3 血浆外泌体的分离

本实验采用超速差速离心法分离血浆外泌体^[7,16]^,分离方法如下:将血浆在室温下以3000 g离心10 min,上清液用0.22 μm孔径过滤器过滤,滤液用70 Ti转子在4 ℃下以120000 g离心3 h,弃去上清液,所得沉淀用磷酸盐缓冲液(PBS, 8 g NaCl、0.2 g KCl、1.44 g Na_2_HPO_3_、0.44 g KH_2_PO_3_、1 L H_2_O, pH 7.4)重悬后再以120000 g离心2 h,沉淀用PBS洗涤,最终得到血浆外泌体。

### 1.4 血浆高丰度蛋白质去除

为了提高低丰度蛋白质的检出率,选用高选择性Top14高丰度蛋白质损耗树脂来去除血浆中丰度最高的14种蛋白质^[14]^。使用Bradford法测定蛋白质浓度后,向样本中加入4倍体积且已在-20 ℃预冷的丙酮,静置过夜;之后于4 ℃、12000 g下离心10 min,收集沉淀,并在真空离心蒸发浓缩器中干燥。

### 1.5 外泌体的表征

#### 1.5.1 透射电镜观察外泌体形态

使用透射电镜对外泌体的形态进行观察,用20 mmol/L三羟甲基氨基甲烷盐酸盐(Tris-HCl, pH 6.8)缓冲液将样本稀释至质量浓度为0.1 μg/μL。在无菌滤纸上滴1滴醋酸铀、2滴无菌水,将碳网盖在样本上静置2 min,用滤纸吸取多余样本,并于室温下晾干。取10 μL醋酸铀溶液负染1 min,用滤纸吸取多余染剂并在无菌水中清洗30 s,最后将样本放置于显微镜下进行观测拍照。

#### 1.5.2 蛋白免疫印迹分析检测外泌体标记蛋白质

取10 μg已测定蛋白质浓度的样本,与1%十二烷基硫酸钠裂解液混合后进行蛋白免疫印迹分析。在进行蛋白质样品电泳时,先在100 V恒压下电泳15 min,然后在200 V恒压下电泳45 min,随之结束电泳。将与十二烷基硫酸钠聚丙烯酰胺凝胶电泳胶大小相近的纤维素膜预先在甲醇中浸泡摇晃约10 min,然后按照由上至下依次为滤纸、凝胶、纤维素膜、滤纸的顺序放在转膜仪上,转膜条件设置为100 mA、10 V持续1 h。转膜结束后,在室温条件下使用PBS配制的5%(质量分数)脱脂奶粉来封闭纤维素膜1 h;随后切下适当分子质量的样本条带,向其中加入相应稀释比例的一抗,在4 ℃摇床中孵育过夜;在室温下使用PBS洗膜3次,每次洗涤10 min,之后向其中加入与一抗种属相符的二抗,室温下孵育1 h;再使用PBS洗膜3次,每次洗涤10 min,加入化学发光HRP底物反应2 min,在暗室进行曝光,使用Image J软件对蛋白质表达量的变化进行灰度分析和比较。目的条带的分子质量:Calreticulin 55 kDa, Flotillin-1 48 kDa, Alix 96 kDa。

#### 1.5.3 纳米粒子跟踪分析(NTA)检测外泌体样本粒径

将外泌体样本用PBS以1∶1000(v/v)的比例进行稀释,使用配备了405 nm激光的纳米颗粒跟踪分析仪来测定外泌体的粒径和浓度,并记录纳米粒子在1 min内的运动轨迹;之后用NTA软件(version 8.02.28)对所记录的视频进行分析,并提供粒子尺寸分布和粒子浓度数值。

### 1.6 样本前处理

在每个样品中均取40 μg蛋白质,并用8 mmol/L尿素溶液定容至60 μL,再向其中加入3 μL重组赖氨酰内切酶(Lys-C),并于37 ℃下酶解2 h。用100 mmol/L Tris-HCl(pH 8.5)将样品稀释4倍,再向其中加入2 μL胰蛋白酶(trypsin),用封口膜包住离心管,在37 ℃温箱中酶解8~18 h。向样本溶液中加入三氟乙酸(TFA)进行酸化(加入后TFA的体积分数为0.5%),选用搭载了C18的200 μL枪头脱盐柱进行脱盐,随后在3000 g下离心1 min,用200 μL乙腈(ACN)、200 μL 0.1%TFA水溶液-ACN(3∶7, v/v)润洗脱盐柱,再用200 μL 0.1% TFA水溶液平衡脱盐柱;之后缓慢加入样品,在3000 g下离心2 min,收集流出样品,用200 μL 0.1%TFA水溶液进行脱盐;随后使用200 μL 0.1%TFA水溶液-ACN(3∶7, v/v)对样品进行洗脱,收集洗脱液。对洗脱后的肽段样本进行液氮速冻处理,之后在真空旋转干燥仪中完全干燥。用100 mmol/L四乙基溴化铵(TEAB)对已干燥的肽段进行复溶,测定肽段浓度,每个样品取等量的20 μg肽段用于标记。使用10标TMT进行标记,然后加入8 μL 5%羟胺(HDX),混匀后于室温下放置15 min以终止反应。将18个样本分成两批次进行分析,每批次均设置一个含有等量各样本组分的内参。使用ACN和0.1%氨水配制梯度洗脱液(ACN的体积分数依次为10%、12.5%、15%、17.5%、20%、22.5%、25%和50%),用C18脱盐柱对0.1%氨水溶解的TMT标记肽段进行脱盐,再使用上述梯度洗脱液对样本进行分级洗脱,并将使用10%ACN及50%ACN洗脱液洗脱的样本混合。最后将上述7个馏分进行真空干燥,备用。

### 1.7 LC-MS/MS分析条件

#### 1.7.1 色谱条件

用0.1%甲酸水溶液将TMT标记的多肽样品复溶,之后以8 μL/min的速率通过自动进样器进入C18色谱柱(2 cm×75 μm, 3 μm)中,然后将C18柱切换到PepMap RSLC C18柱。流动相A为0.1%甲酸水溶液,流动相B为乙腈。梯度洗脱程序:0~15 min, 2%B~25%B; 15~25 min, 25%B~50%B; 25~28 min, 50%B~98%B。流速300 nL/min;柱温40 ℃;进样量10 μL。

#### 1.7.2 质谱条件

离子化模式为正离子模式,扫描模式为全扫描(*m/z* 300~1500),扫描频率为10000 Da/s,数据采集模式为数据依赖型采集(data-dependent acquisition, DDA)。一级质谱扫描范围为*m/z* 350~1800,分辨率为120000;选取一级质谱图中信号最强的母离子进行二级裂解,二级扫描的分辨率为50000,碎裂模式为高能碰撞解离(HCD),归一化碰撞能(NCE)设置为37%。

### 1.8 质谱数据检索分析

利用Proteome Discoverer 2.2软件的SEQUEST搜索引擎功能对得到的质谱原始数据进行检索,检索数据库为UniProt人源数据库Human. fasta(2017年10月下载)。检索参数如下:胰酶特异性全酶切,允许两个漏切;固定修饰包括半胱氨酸的烷基化、肽段N端或赖氨酸的TMT修饰;可变修饰为甲硫氨酸的氧化;母离子和碎片离子的质量误差分别为1×10^-5^ Da和0.02 Da;蛋白质和肽段谱图鉴定的错误发现率(false discovery rate, FDR)小于1%;对MS/MS扫描报告中不同离子通道的肽段丰度进行标准化^[17]^。蛋白质鉴定要求每个蛋白质组至少有一个唯一肽段,蛋白质定量采用TMT报告离子定量,并利用内参对不同TMT实验之间的报告离子强度进行归一化。

### 1.9 数据分析及生物信息学分析

蛋白质组的所有统计分析均在RStudio环境(v1.0.143)中使用R(v3.6.3)进行。使用log_2_变换后的双尾*t*检验来分析差异表达蛋白质,*P*采用多重检验校正方法(benjaminiand Hochberg, BH)进行调整^[18]^。在对热图进行可视化分析之前,根据蛋白质的*Z*评分(*Z*-score)对蛋白质的表达水平进行聚类分析。以*P*<0.05和差异倍数(fold change, FC)>1.2为条件,筛选HC和PD组之间的差异表达蛋白质。通过韦恩图对鉴定到的血浆外泌体蛋白质与ExoCarta外泌体数据库(http://www.exocarta.org)进行比较;使用MAGeCKFlute R包中的enrichment analyze函数进行京都基因和基因组百科全书(KEGG)通路和基因本体论(GO)富集分析,并使用MAGeCKFlute中的函数对富集路径进行可视化^[19]^。

## 2 结果与讨论

### 2.1 血浆外泌体的表征与鉴定

在透射电镜下可观察到外泌体典型的双侧凹陷双层膜结构,呈圆形或椭圆形囊泡状,如图1a和1b所示。囊泡可单一分布也可聚集成群叠加分布,尺寸约为100 nm,结构完整。

**图1:**
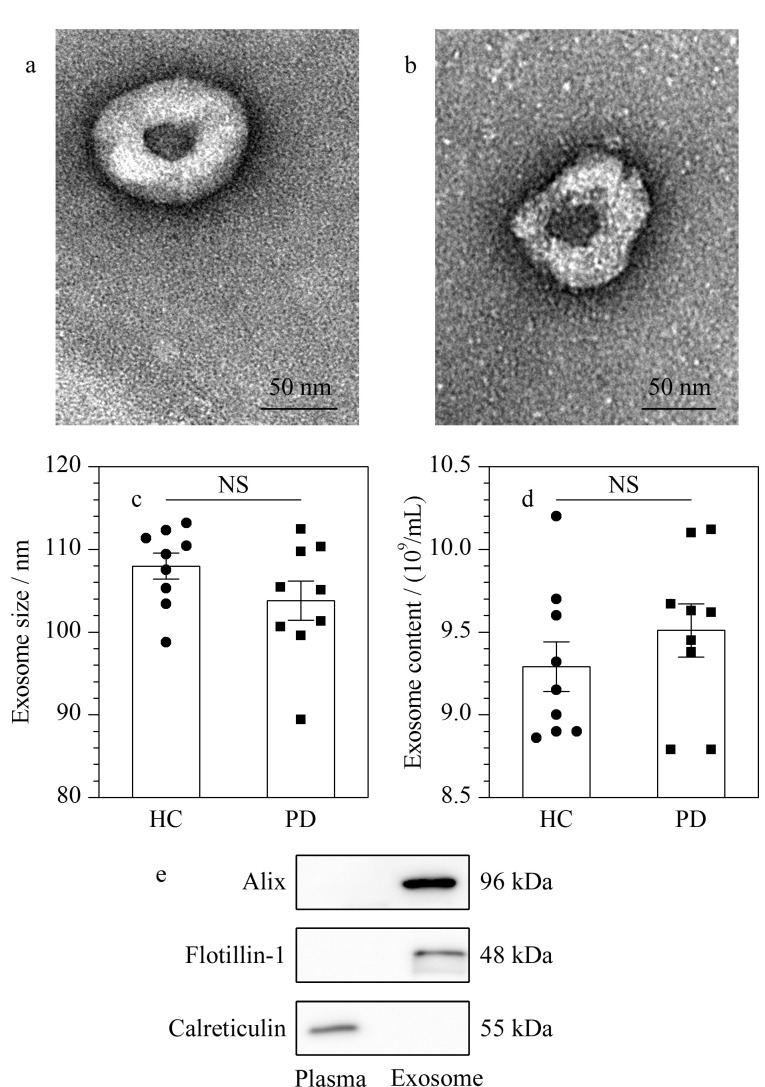
血浆外泌体表征

利用NTA软件对外泌体样本的粒径及含量进行分析,如图1c所示,经过1000倍稀释后,HC组与PD组的外泌体粒径分别为107.96±1.57 nm和103.80±2.35 nm,均在外泌体的粒径范围之内,且两组外泌体粒径的差异无统计学意义(*P*=0.35)。如图1d所示,HC组与PD组外泌体的颗粒含量分别为9.29×10^9^个/mL和9.51×10^9^个/mL,两组外泌体含量的差异无统计学意义(*P*=0.16)。使用免疫印迹法检测外泌体标志性蛋白质(即外泌体阳性蛋白质)Alix、Flotillin-1和内质网标志性蛋白质(即外泌体阴性蛋白质)Calreticulin的表达情况。如图1e所示,通过差速超速离心法富集的人血浆外泌体中,Alix、Flotillin-1呈高表达,而Calreticulin的表达量明显减少,甚至消失。此外,将鉴定到的外泌体蛋白质与ExoCarta外泌体数据库进行比对分析,大部分蛋白质均可在外泌体数据库中检索到,如图2所示。以上结果均验证了该外泌体富集方法的可靠性,同时证明了所获得的外泌体具有较高的纯度。

**图2:**
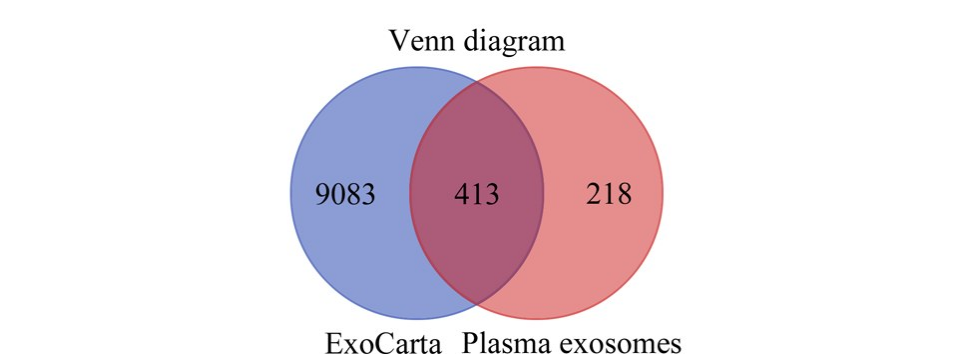
血浆外泌体蛋白质与ExoCarta外泌体数据库比对情况

### 2.2 PD患者与HC的血浆及血浆外泌体蛋白质质谱分析

本研究通过LC-MS/MS技术对PD患者和HC的血浆及血浆外泌体进行基于TMT的定量蛋白质组学分析,并比较了血浆和血浆外泌体两种样本的蛋白质质谱图。在血浆和血浆外泌体样本中分别鉴定到759和650个蛋白质,定量到724和611个蛋白质。对定量到的蛋白质表达值进行标准化处理,标准化前后的比值分布以箱线图表示。如图3所示,标准化后的数据更趋近于中心位置,样品间的强度分布差异更小,有利于后续数据分析。

**图3:**
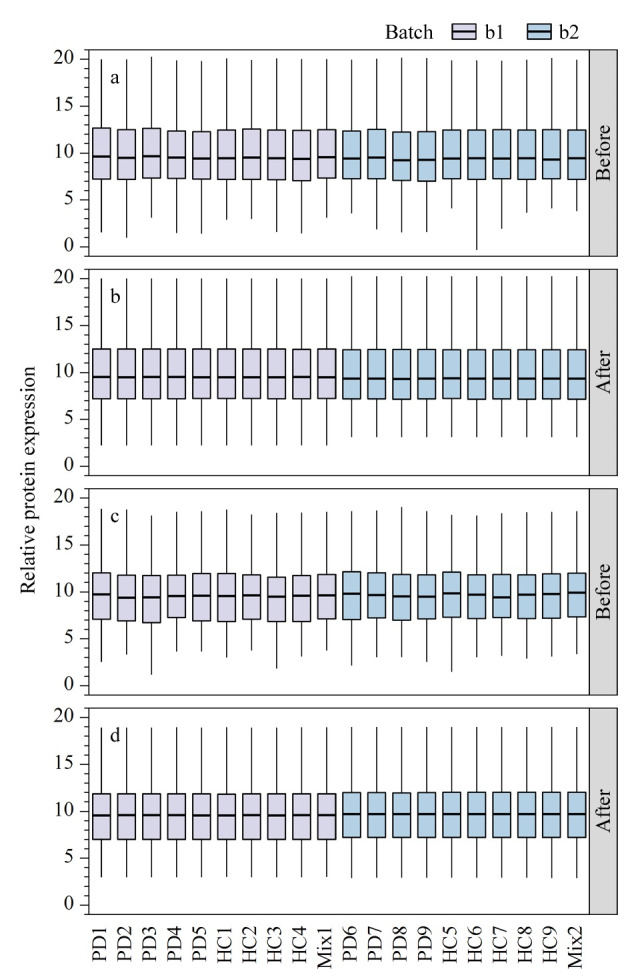
定量到的(a, b)血浆蛋白质和(c, d)血浆外泌体蛋白质在标准化处理前后的比值分布

基于PD组与HC组经标准化处理后的蛋白质丰度,做非配对*t*检验,并对*t*检验所得到的*P*进行多重检验校正。在血浆蛋白质的鉴定中,由于血浆中存在大量高丰度蛋白质,导致血浆低丰度蛋白质被掩盖,降低了质谱中可鉴定的蛋白质数量。因此,本研究采用高丰度蛋白损耗树脂来去除血浆中丰度最高的14种蛋白质。同时,在每批TMT实验中均设置了同质量的内参,以保证组间定量结果的可比性。以|log_2_FC|>0.26和*P*<0.05为筛选条件,在PD组与HC组血浆样本中共筛选到11个显著差异表达蛋白质,其中5个蛋白质表达上调,6个蛋白质表达下调;而在血浆外泌体样本中共筛选到13个显著差异表达蛋白质,其中6个蛋白质表达上调,7个蛋白质下调。由此可见,血浆外泌体中可以筛选到更多的差异表达蛋白质。如表2所示,人血浆激肽释放酶(KLKB1)、胞外5'-核苷酸酶(NT5E)、*α*-*N*-乙酰葡糖胺糖苷酶(NAGLU)、人前列腺转谷氨酰胺酶(TGM4)、凝血因子Ⅷ (F8)、三磷酸腺苷酶蛋白酶体26S亚基4 (PSMC4)、脂多糖结合蛋白受体(CD14)、碳酸酐酶Ⅳ(CA4)、分泌型磷蛋白2 (SPP2)、脂多糖结合蛋白(LBP)均可在外泌体蛋白质数据库中检索到。如图4所示,与血浆蛋白质分布相比,PD组与HC组血浆外泌体样本的蛋白质分布差异度更大。因此,对血浆外泌体样本进行高通量质谱分析有助于发现与疾病相关的新蛋白质。

**表2:** PD组与HC组血浆和血浆外泌体中的差异表达蛋白质

Plasma					Plasma exosome				
Accession^*^	Description	log_2_ FC (PD/HC)	*P*		Accession		Description	log_2_ FC (PD/HC)	*P*
A0A075B6R9	IGKV2D-24	-0.34	0.041		A0A0A0MT89		IGKJ1	0.63	0.032
A0A0G2JL56	HLA-A	0.29	0.006		A0A0B4J1X5		IGHV3-74	0.35	0.038
A6NJZ7	RIMBP3C	0.70	0.044		H0YAC1		KLKB1	0.47	0.025
K7ES70	MFAP4	0.38	0.045		P00451		F8	0.51	0.049
O15335	CHAD	-0.30	0.009		P01715		IGLV3-1	-0.73	0.008
P03973	SLPI	-0.49	0.024		P08571		CD14	0.27	0.012
P08514	ITGA2B	0.37	0.033		P18428		LBP	0.49	0.032
P14780	MMP9	-0.34	0.009		P21589		NT5E	0.36	0.035
P20023	CR2	-0.34	0.022		P22748		CA4	0.45	0.021
P58335-4	ANTXR2	-0.29	0.016		P43686		PSMC4	0.30	0.034
Q6KB66-3	KRT80	0.36	0.023		P49221		TGM4	0.44	0.021
					P54802		NAGLU	0.37	0.017
					Q13103		SPP2	0.31	0.043

* Uniprot accession; IGKV2D-24: immunoglobulin kappa variable 2D-24 (non-functional, fragment); HLA-A: HLA class Ⅰ histocompatibility antigen; RIMBP3C: Rab3 binding protein 3C; MFAP4: microfibril-associated glycoprotein 4; CHAD: chondroadherin; SLPI: antileukoproteinase; ITGA2B: integrin alpha-Ⅱb; MMP9: matrix metalloproteinase-9; CR2: complement receptor type 2; ANTXR2: isoform 4 of anthrax toxin receptor 2; KRT80: isoform 3 of Keratin, type Ⅱ cytoskeletal 80; IGKJ1: immunoglobulin kappa joining 1; IGHV3-74: immunoglobulin heavy variable 3-74; KLKB1: plasma kallikrein (fragment); F8: coagulation factor Ⅷ; IGLV3-1: immunoglobulin lambda variable 3-1; CD14: monocyte differentiation antigen 14; LBP: lipopolysaccharide-binding protein; NT5E: 5'-nucleotidase; CA4: carbonic anhydrase 4; PSMC4: 26S proteasome regulatory subunit 6B; TGM4: protein-glutamine gamma-glutamyltransferase 4; NAGLU: *α*-*N*-acetylglucosaminidase; SPP2: secreted phosphoprotein 2.

**图4:**
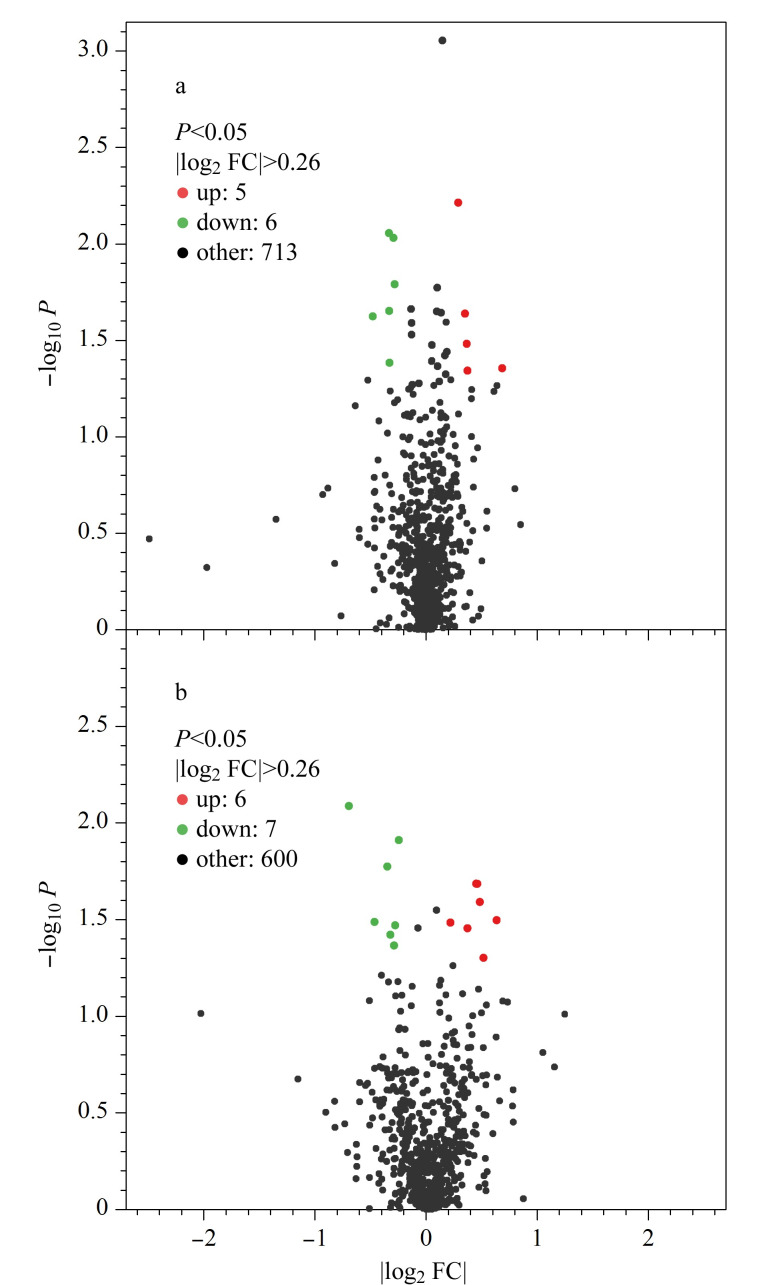
PD组与HC组(a)血浆及(b)血浆外泌体差异表达蛋白质分布图

据文献[20⇓-22]报道,NAGLU、NT5E等蛋白质的差异表达不仅会增加PD的患病风险,还会参与PD的病理过程,因此这些蛋白质具有成为新型PD标志物的潜力。NT5E是参与调节细胞稳态、应激反应、损伤和疾病等过程的重要水解酶,其可催化磷酸腺苷(AMP)形成胞外腺苷^[22⇓-24]^。在PD小鼠脑组织模型中,NT5E上调并激活腺苷2A(A2A)受体相关的腺苷通路;敲除NT5E能够抑制PD模型中A2A受体的激活和上调,并发挥保护多巴胺能神经元和减轻小鼠运动障碍的作用。进一步研究发现,NT5E可促进PD小胶质细胞的激活和炎症因子的释放^[20]^。NAGLU是一种降解硫酸肝素糖胺聚糖(GAGs)所需的溶酶体酶^[25,26]^,从遗传学角度来看,NAGLU的多态性(rs2071046)与PD的风险增加有关。Winder-Rhodes等^[27]^对926名PD患者和2308名对照者进行了研究,发现C等位基因纯合度的比值比为1.32。除此之外,Hamano等^[21]^发现ⅢB型黏多糖贮积病(MPSⅢB)患者的脑部区域内存在大量磷酸化的*α*-syn,从病理角度说明NAGLU可能与*α*-syn的生成、代谢及聚集密切相关。LBP是一种在肝脏中产生并可进入血液循环的糖蛋白,它可以促进巨噬细胞与脂多糖的结合,导致炎症细胞因子分泌增加^[28]^。已有研究表明,血浆中的LBP水平可以反映PD患者的患病风险、运动症状严重程度及疾病进展^[29]^。

### 2.3 GO及KEGG通路富集分析

本研究以蛋白质表达差异大小为基础排序,采用基因集富集分析GSEA对定量到的所有差异表达蛋白质进行功能富集分析^[30]^。GSEA有助于了解所有差异基因的总体变化趋势,可以避免一些变化微弱却具有效力的蛋白质被过滤掉,发现更多与疾病相关的生物学通路。本研究对血浆和血浆外泌体样本均进行了基于GSEA的GO和KEGG分析。在GO分析中,依据*P*<0.05将基因本体论-生物学过程(GO-BP)、基因本体论-细胞组分(GO-CC)、基因本体论-细胞功能(GO-MF)中的前10名富集功能通路进行展示,结果如图5所示。

**图5:**
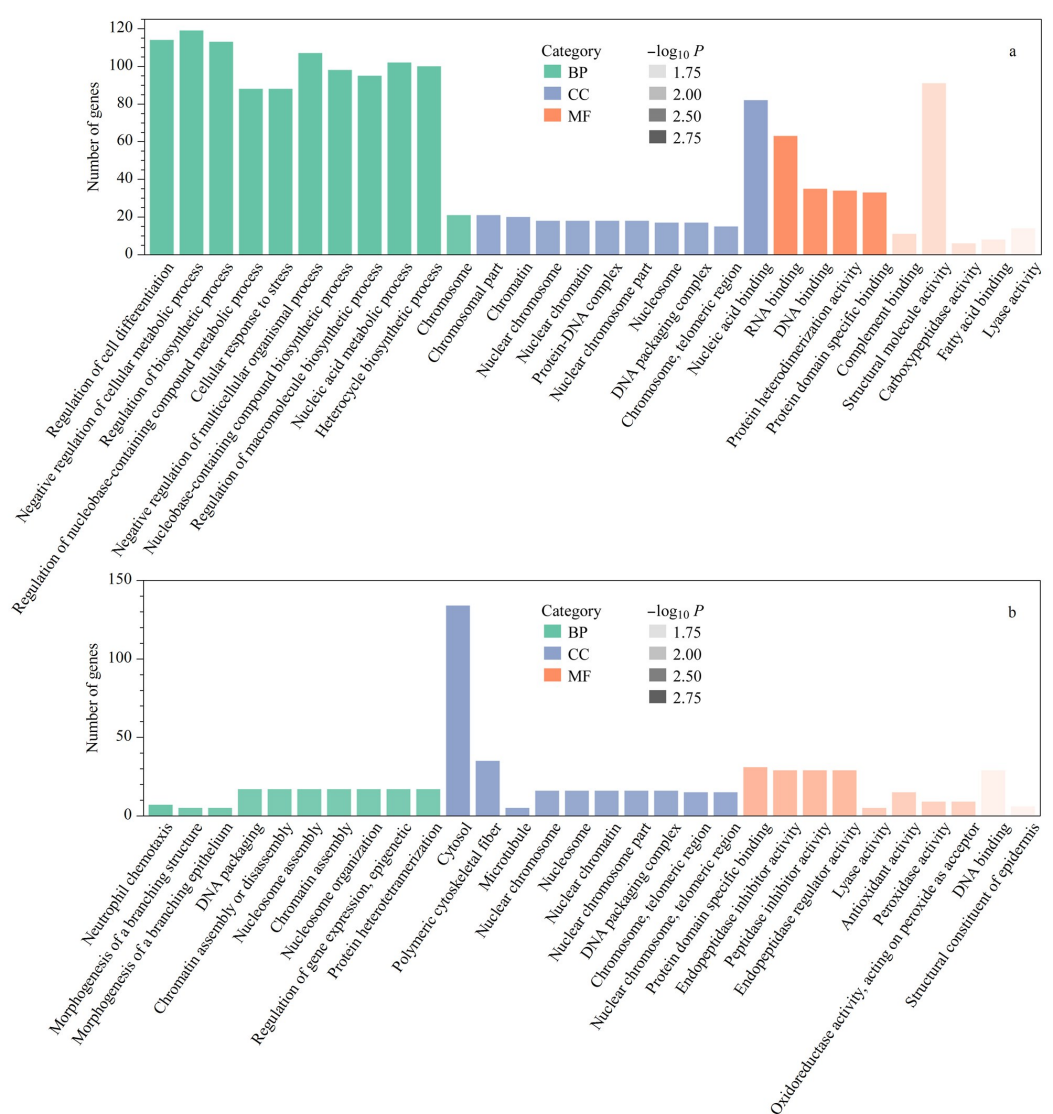
PD组与HC组(a)血浆和(b)血浆外泌体样本的差异表达蛋白质GO分析

依据GO-CC分析,血浆富集蛋白质主要定位于细胞核,而血浆外泌体富集蛋白质主要定位于细胞质中。由于外泌体成分主要为各种细胞外分泌的产物,其内含有大量细胞浆内物质,这与GO分析中的细胞组成结果一致。GO-MF分析显示,血浆差异表达蛋白质的分子功能主要富集为RNA、DNA结合及补体结合等,而血浆外泌体差异表达蛋白质的分子功能主要富集为抗氧化作用和氧化还原酶活性等。由此可见,外泌体差异表达蛋白质富集到的分子功能更具有疾病特异性。研究表明,氧化应激损伤在PD中可起到重要作用。多巴胺能神经元有巨大、无髓鞘的轴突并可产生超过100万的突触,其对能量的需求较其他神经元更多。PD中多巴胺能神经元的能量平衡被打破后,会导致其需氧量增加,而这种能量的不平衡又进一步加重了氧化应激损伤,致使黑质纹状体内大量多巴胺能神经元的死亡丢失^[31,32]^。

最后,对不同样本差异表达蛋白质的KEGG富集通路进行分析(图6)。血浆样品富集通路有溶酶体途径(lysosome)、细胞老化(cellular senescence)及内质网反应(protein processing in endoplasmic reticulum)等,而血浆外泌体样本差异表达蛋白质主要富集为细胞因子途径(chemokine signaling pathway)和细胞因子受体间相互作用(cytokine-cytokine receptor interaction)等。溶酶体是自噬途径的最终作用场所,负责错误折叠蛋白质及受损细胞器的清除。

**图6:**
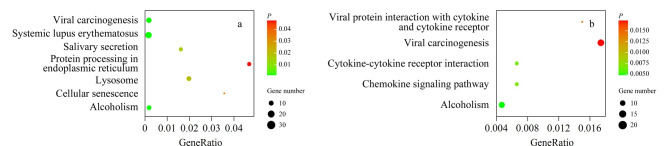
PD组与HC组(a)血浆样本与(b)血浆外泌体样本的KEGG通路分析

因此,溶酶体介导的自噬在PD病理过程中,尤其是维持黑质神经元功能中发挥重要作用^[33]^。研究表明,野生型*α*-syn可通过分子伴侣介导在热激蛋白70(Hsc70)的协助下转运至溶酶体并被降解,而构象发生变化的病理性*α*-syn不能被降解,这是因为病理性*α*-syn与溶酶体膜上的特异性受体有特别高的亲和力,该类受体对自噬途径具有调控作用。病理性*α*-syn与受体结合后可阻断溶酶体摄入*α*-syn,并抑制其和其他底物的降解,造成胞内蛋白质的异常聚集^[34,35]^。此外,溶酶体三磷酸腺苷酶(ATPase, ATPl3A2)的突变也是引起PARK9相关帕金森病的主要原因。ATPl3A2是编码溶酶体膜蛋白质的主要基因,其突变可导致PD病人的自噬功能受损及*α*-syn在黑质纹状体中的聚集^[36]^。

与包含较多无关信息的血浆样本相比,对特异性更强的血浆外泌体样本进行分析可以更加直观地反映出机体和疾病的不同状态。分析其原因可能是外泌体通过内吞途径起源于核内体,而核内体早期起源于内质网、高尔基体和线粒体等细胞内膜系统,外泌体通过两次质膜内陷从晚期核内体中分离出来,因此其携带了大量的母细胞信息,并通过融合、受体相互作用或内吞作用等释放至胞外^[2]^。在PD进展过程中,外泌体不仅可以向远处播散*α*-syn,而且会通过分泌其他生物分子(如炎症因子等)直接或间接地干扰邻近神经元或胶质细胞的病理、生理过程。因此,外泌体内含有更多与PD相关的生物活性分子,对其进行高通量组学分析更有利于发现与疾病相关的蛋白质、病理机制以及新型疾病标志物。

然而本研究也存在一定的局限性。首先样本量较少,尽管发现了一些新型的、与PD相关的血浆及血浆外泌体蛋白质,但缺乏大样本量的验证,不能很好地评价其对PD的诊断价值;其次,PD为中枢神经系统的退行性疾病,由于血脑屏障的存在,本研究中发现的差异表达蛋白质并不能完全反应PD脑组织中的蛋白质表达差异,需要进一步在动物及细胞模型中进行功能验证;最后,尽管本研究发现血浆外泌体蛋白质富集通路或富集蛋白质功能较血浆蛋白质更具有疾病特异性,但与中枢神经系统来源外泌体相比,其特异性仍偏低,之后可以对中枢神经系统来源外泌体进行高通量质谱分析,以获取更多与疾病相关性更强的生物学信息。

## 3 结论

本研究基于TMT的定量蛋白质组学技术对PD患者和HC的血浆及血浆外泌体样本进行差异表达蛋白质筛选和GSEA生物学信息分析,研究结果将有助于进一步了解PD的疾病机制。尽管血浆蛋白质和血浆外泌体蛋白质均在PD中出现了明显的差异表达,但与血浆样本蛋白质组分析相比,血浆外泌体蛋白质组学分析可以获得更多有价值的差异表达蛋白质及生物学信息。因此,本研究不仅证实了外泌体是作为新型PD标志物及诊疗靶点的良好研究载体,也论证了蛋白质组学技术在疾病标志物研发中的重要作用。
